# Compassion-focused therapy (CFT) for the reduction of the self-stigma of mental disorders: the COMpassion for Psychiatric disorders, Autism and Self-Stigma (COMPASS) study protocol for a randomized controlled study

**DOI:** 10.1186/s13063-023-07393-y

**Published:** 2023-06-12

**Authors:** M. Riebel, O. Rohmer, E. Charles, F. Lefebvre, S. Weibel, L. Weiner

**Affiliations:** 1 Laboratoire de Psychologie des Cognitions (Unistra), Université de Strasbourg, 12 rue goethe, 67000 Strasbourg, France; 2 Pôle de Psychiatrie, Santé Mentale et Addictologie, Hôpitaux Universitaires de Strasbourg, 1 place de l’hôpital, 67000 Strasbourg, France; 3 Groupe Méthode en Recherche Clinique (GMRC), Strasbourg University Hospitals (SUH), Strasbourg, France

**Keywords:** Compassion-focused therapy (CFT), Randomized controlled trial (RCT), Self-stigma, Cognitive-behavioral therapy, Shame, Autism spectrum disorder, Bipolar disorder, Schizophrenia, Borderline personality disorder, Depression, Ending Self-Stigma, Severe mental illness (SMI)

## Abstract

**Background:**

People with mental disorders face frequent stigmatizing attitudes and behaviors from others. Importantly, they can internalize such negative attitudes and thus self-stigmatize. Self-stigma is involved in diminished coping skills leading to social avoidance and difficulties in adhering to care. Reducing self-stigma and its emotional corollary, shame, is thus crucial to attenuate the negative outcomes associated with mental illness. Compassion-focused therapy (CFT) is a third-wave cognitive behavioral therapy that targets shame reduction and hostile self-to-self relationship and allows for symptom improvement while increasing self-compassion. Although shame is a prominent part of the concept of self-stigma, the efficacy of CFT has never been evaluated in individuals with high levels of self-stigma. The purpose of this study is to evaluate the efficacy and acceptability of a group-based CFT program on self-stigma, compared to a psychoeducation program for self-stigma (Ending Self-Stigma) and to treatment as usual (TAU). We hypothesize that diminished shame and emotional dysregulation and increased self-compassion will mediate the relationship between self-stigma improvements post-therapy in the experimental group.

**Methods:**

This seven-center trial will involve 336 participants diagnosed with a severe mental illness and/or autism spectrum disorder and reporting high levels of self-stigma. Participants will be randomized into one of three treatment arms: 12 week-treatment of compassion-focused therapy (experimental arm), 12 week-treatment of Psychoeducation (active control arm), and TAU (treatment as usual—passive control arm). The primary outcome is the decrease of self-stigma scores on a self-report scale, i.e., ISMI, at 12 weeks. Secondary endpoints include sustainability of self-stigma scores (ISMI) and self-reported scores regarding target psychological dimensions, e.g., shame and emotional regulation, social functioning, and psychiatric symptoms. Assessments are scheduled at pretreatment, post-treatment (at 12 weeks), and at 6-month follow-up. Acceptability will be evaluated via (i) the Credibility and Expectancy Questionnaire at T0, (ii) the Consumer Satisfaction Questionnaire for Psychotherapeutic Services posttreatment and at 6-month follow-up, (iii) attendance, and (iv) dropout rates.

**Discussion:**

This study will evaluate the potential efficacy and acceptability of a group-based CFT program on the decrease of self-stigma and thereby contribute to the continuing development of evidence-based therapeutic interventions for the internalized stigma of mental and neurodevelopmental disorders.

**Trial registration:**

ClinicalTrials.gov NCT05698589. Registered on January 26, 2023

## Administrative information

Note: The numbers in curly brackets in this protocol refer to the SPIRIT checklist item numbers. The order of the items has been modified to group similar items (see http://www.equator-network.org/reporting-guidelines/spirit-2013-statement-defining-standard-protocol-items-for-clinical-trials/).

**Table:** 

Title {1}	Compassion-focused therapy (CFT) for the reduction of the internalized stigma of mental disorders: a multi-center, prospective, randomized, controlled study
Trial registration {2a and 2b}	Registered on January 26, 2023: clinicaltrials.gov NCT05698589
Protocol version {3}	Version 1 of 01-3-2022
Funding {4}	Funding to finance this study has been requested after a public invitation to tender from the General Directorate for Healthcare Provision (DGOS) (national PHRC).
Author details {5a}	M. Riebel: Laboratoire de Psychologie des Cognitions (Unistra), France O. Rohmer: Laboratoire de Psychologie des Cognitions (Unistra), France E. Charles: Strasbourg University Hospital (SUH), France S. Weibel: Strasbourg University Hospital (SUH), France L. Weiner: Strasbourg University Hospital (SUH), France; Laboratoire de Psychologie des Cognitions (Unistra), France
Name and contact information for the trial sponsor {5b}	Strasbourg University Hospital (SUH) 1, place de l’Hôpital, F-67 091 STRASBOURG cedex DRCI@chru-strasbourg.fr
Role of sponsor {5c}	The funders played no role in the design of the study; collection, analysis, and interpretation of the data; and writing the manuscript.

## Introduction

### Background and rationale {6a}

Severe mental illness (SMI) is defined as a chronic mental disorder that results in severe dysfunction interfering with or substantially limiting one or more major life activities [1]. The definition proposed by the US National Institute of Mental Health includes three dimensions: diagnosis, chronicity, and associated disability [1]. Examples of disorders categorized as SMI include psychotic disorders, mood disorders (i.e., bipolar disorder and recurrent depression), and personality disorders (e.g., borderline personality disorder). The World Mental Health (WMH) Surveys estimate that the annual prevalence of SMI is between 4 and 6.8% of adults [2]. Despite the existence of pharmacological and psychological treatments with proven efficacy in reducing symptoms, the social inclusion of people with SMI remains dramatically reduced.

It is now widely recognized that factors other than symptoms of the condition and adverse effects of pharmacological treatments can interfere with the prognosis and the social inclusion of people with psychiatric disorders. Specifically, stigmatizing attitudes and behaviors that people with SMI face from others can negatively impact the course of their illness [3–5]. In response to stigmatizing behaviors, they tend to isolate themselves and avoid medical care, as well as social and professional situations, with the risk of hindering care and the overall process of personal recovery and integration into society [6]. Relatedly, SMI is the first cause of occupational disability as well as one of the first causes of unemployment [7] and work absenteeism in Europe [8].

The internalization of stigma, i.e., self-stigma, is thought to be one of the main causes of the reduced social inclusion of people with SMI [9–11]. Self-stigma is defined as the process by which an individual internalizes negative messages about their own social group and applies them to themselves [12]. This phenomenon, which is strongly associated with negative emotions (i.e., shame) [13], self-critical thoughts, and a significant fear of public stigma—that is, stigmatizing attitudes and behaviors from others [3]—prevents individuals from fulfilling and pursuing their life goals [14]. In particular, self-stigma is involved in diminished coping skills that lead to social avoidance and difficulties in adhering to care [7]. Considered as the affective component of self-stigma [13], shame is highly associated with negative self-evaluation and self-criticism, greater severity of clinical presentations, and impeding treatment [15–17]. In addition to increasing the risk of psychopathology, shame can thus be a hindrance to the effectiveness of treatments as it influences the expression of symptoms and the patient’s ability to disclose painful information and to seek help, and it can promote several types of avoidance, such as dissociation and therapy drop-out [16].

Given the relationship between self-stigma and personal recovery in people with SMI, a growing number of studies have focused on this issue in recent years. For instance, a recent systematic review reported high levels of self-reported self-stigma in people with SMI but also in neurodevelopmental conditions such as autism spectrum disorder (ASD) [18]. In addition, recent meta-analyses and systematic reviews have highlighted the negative consequences of self-stigma on multiple aspects associated with the quality of life and the well-being of patients. For example, self-stigma in SMI and ASD is related to lower self-esteem and a reduced feeling of self-efficacy [19, 20], as well as lower levels of quality of life, increased risk of depressive and anxious symptoms, and increased hopelessness [19, 21, 22]. From a personal recovery perspective, self-stigma is linked to impaired social functioning, high levels of social withdrawal, high rates of unemployment, and reduced motivation to engage in pro-health behaviors, i.e., poorer physical health and poorer treatment adherence [18, 23–25]. Reducing self-stigma and its emotional corollary, i.e., shame, is thus crucial to attenuate the disability associated with mental illness and improve the quality of life and social inclusion of people with SMI and ASD.

Over the last decade, research focusing on the social inclusion and personal recovery of people with SMI and ASD has focused increasingly on psychosocial treatments aimed at reducing self-stigma. However, psychological treatments for the reduction of self-stigma remain rare, and recent meta-analyses and systematic reviews have highlighted several methodological pitfalls in existing studies assessing the feasibility and efficacy of interventions (e.g., lack of randomized controlled studies) [26, 27]

Interventions aimed at reducing self-stigma can be classified into four categories (i) psychoeducation, (ii) second-wave cognitive behavioral therapies (CBT) aiming to change beliefs (internalized stereotypes) related to mental illness, (iii) interventions targeting the disclosure of one’s mental illness, and (iv) multi-component interventions. On average, the existing interventions are composed of 10 sessions and are carried out in groups [26–29].

Among the interventions designed to reduce self-stigma in people with SMI, group psychoeducation has been the most studied [26–28]. In particular, the Ending Self-Stigma (ESS) program [12, 29, 30] is among the rare interventions whose efficacy has been tested via a randomized controlled trial (RCT) [30]. The ESS program is a 9-session manualized group intervention during which participants learn skills aimed at reducing self-stigma. Sessions consist of learning cognitive strategies (e.g., identifying and changing self-stigmatizing thoughts) and problem solving. In their study, Lucksted et al. [30] compared this intervention to treatment as usual (TAU) and found an immediate improvement in the alienation and stigma resistance subscales of the Internalized Stigma of Mental Illness Scale (ISMI) [31] but no improvement on the total self-stigma score. Moreover, the effects were modest, failed to be significant at 6 months of post-treatment follow-up, and the authors reported a very high drop-out rate and low attendance (only 43% of participants attended at least 7 sessions). Similar results suggesting the high attrition rate of the ESS have been reported in a more recent study [12]. The latter indicates no superior effects of ESS compared to a nonspecific health and well-being program. These different trials emphasize the limitations of the ESS (e.g., the attrition rates, the inconclusive results when the ESS was compared to an active control group, and the lack of sustainability of self-stigma outcomes at follow-up). Given that the affective component of self-stigma, i.e., shame, was not targeted by the ESS, we hypothesize that high levels of shame lingered post-therapy leading both to social avoidance and high rates of drop-out.

Overall, despite the aforementioned limitations, psychoeducation programs such as the ESS [30] remain recommended in recent meta-analyses and systematic reviews as they cumulated the highest level of evidence for reducing self-stigma associated with SMI. The aim of our compassion-focused intervention is to address the main limitations of more traditional approaches that do not address the shame associated with self-stigma. To do so, unlike traditional CBT and psychoeducation programs, our intervention aims to directly target the affective component of self-stigma, i.e., shame [13, 17], through compassion-focused therapy (CFT) [16, 32].

CFT is part of third-wave CBT. Importantly, CFT specifically targets shame reduction and hostile self-to-self relationships and allows for symptom improvement [33] while increasing self-compassion, a major resilience factor [34]. In CFT, compassion is defined as a motivation that orients to *a sensitivity to suffering in self and others with a commitment to try to alleviate and prevent it* [35]. CFT builds on traditional CBT principles and blends empirical knowledge from affective neuroscience, social and developmental psychology, and mindfulness. CFT has gathered a large body of evidence in the treatment of shame and self-blame in a wide range of clinical settings [e.g., [16, 33, 36–40]].

Although CFT focuses on the treatment of shame-related difficulties, few studies have focused on the relationship between self-stigma and self-compassion. In a recent study, Wong et al. [11] provided a theoretical framework emphasizing the potential benefits of CFT for self-stigma. According to the authors, self-compassion could buffer the negative effects of stigma by facilitating more social resources and increasing the willingness to ask for help [11]. Therefore, given its focus on the psychological processes and affective aspects of self-stigma, CFT may be more effective than existing interventions in the reduction of self-stigma and shame [11]. More specifically, CFT targets emotions and attitudes (i.e., shame and avoidance) directly and could address the gaps of existing interventions, which focus mainly on cognitive (beliefs and stereotypes) changes. Supportive of this view, numerous social psychology studies suggest that it is necessary to target the affective dimensions of stigmatizing attitudes (i.e., shame) to achieve lasting behavioral changes [4, 41, 42]. Indeed, emotions and feelings are better predictors of change in behaviors than beliefs (for reviews, see [43, 44]).

The purpose of our study is to evaluate the efficacy and acceptability of a transdiagnostic CFT group for self-stigma compared to ESS [30] and treatment as usual (TAU). CFT has been found to be feasible and to reduce shame in SMI including personality disorders [37], patients with psychosis [45, 46], as a transdiagnostic group [47] and, in a pilot study led by our group, in patients presenting with SMI and ASD [48]. However, CFT has not been used to specifically target self-stigma and shame in this population. Our adaptation of CFT aims to address the limitations of existing self-stigma reduction programs such as the ESS [e.g., [30]], i.e., (i) lack of superior effects when compared to active control interventions, (ii) low attendance and high dropout rates, and (iii) lack of long-term effects, which can be attributed to the fact that interventions aimed at deconstructing beliefs and did not target shame.

#### Objectives {7}

The primary objective of our study is to evaluate the efficacy of a 12-week CFT group in decreasing self-stigma associated with severe psychiatric disorders and ASD by comparing its immediate effects to the effects of the Ending Self-Stigma (ESS) psychoeducation program [29] and to TAU.

The secondary objectives of our study are as follows:

- 1. To evaluate the maintenance of effects on self-stigma scores in the three groups of treatment (CFT, ESS, and TAU) at 6-month follow-up (V2)
- 2. To assess the effects of treatment (CFT, ESS, and TAU) at baseline (V0), post-treatment (V1), and at 6 months follow-up (V2) on target psychological dimensions (shame, self-compassion, emotional regulation, automatic avoidance tendencies toward mental illness)
- 3. To assess the effects of treatment (CFT, ESS, and TAU) at baseline (V0), post-treatment (V1), and at 6 months follow-up (V2), on social functioning and psychiatric measures (psychiatric symptoms, depression, anxiety and stress, social functioning, access to care, recovery, functional remission)
- 4. To assess the psychological factors mediating (shame, self-compassion, and emotional dysregulation) the efficacy of CFT on the self-stigma measure (V1)
- 5. To evaluate the acceptability of the active interventions (CFT and ESS): pretreatment (V0) via a treatment credibility measure, during treatment via attrition rate, session attendance and group cohesion, and post-treatment (V1) via patient satisfaction and treatment side effects In addition, in the coordinating center, qualitative interviews will assess the acceptability of the active interventions.

### Trial design {8}

This project is a seven-center, prospective, randomized, parallel-group, controlled superiority trial with three arms: an experimental arm with 12 CFT group sessions; an active control arm with 12 psychoeducation-ESS group sessions, and a passive control arm consisting of TAU. The allocation ratio is 1:1.

## Methods

### Study setting {9}

The COMPASS trial will be performed in 7 French national centers (Strasbourg University Hospital, Psychotherapeutic Center of Nancy, le Vinatier Hospital, Montpellier University Hospital, Bordeaux University Hospital, Clermont-Ferrand University Hospital, Reims University Hospital). Patients will be recruited by psychiatrists or psychologists in the seven centers, where data will be collected during the trial.

### Participants

#### Eligibility criteria {10}

The included centers are nationally recognized as central coordinators of care (within a recovery of mental disorders model) in their respective regions.

## Inclusion criteria

Patients must meet the following criteria to be eligible for the study:

- Patients≥ 18 years of age
- Patient affiliated to a social health insurance plan (beneficiary or beneficiary’s family)
- Patient with one or several diagnoses of chronic psychiatric disorder (schizophrenia, schizoaffective disorder, bipolar disorder, recurrent major depression, borderline personality disorder) or a neurodevelopmental disorder (autism spectrum disorder) treated as an outpatient or in a day hospital
- CGI-Severity score < 6, assessed by the psychiatrist [49]
- ISMI score indicating moderate to high self-stigma (> 2) [50]

## Non-inclusion criteria

They will not be eligible for the study if any of the following applies:

- Patient in an exclusion period determined by a previous or ongoing study
- Patient participating in an interventional study involving psychotherapy or an experimental drug
- Patient in acute episode of their disorder according to the CGI Severity score
- Patient in a medical emergency or immediate life-threatening situation
- Patients with an intellectual disability (IQ < 70) estimated via the fNART [51]
- Legal issues: care under constraint or patient deprived of freedom because of a judicial - measure
- Patient who does not speak and read French sufficiently

## Inclusion criteria for individuals who will perform the interventions

- The facilitators of both psychotherapies are mental health professionals or peer support worker.
- In each psychotherapy group of 8 patients, one of the facilitators is a senior psychologist.

Senior psychologists are trained in either (i) CFT for the experimental arm or (ii) the ESS program for the active control arm

### Who will take informed consent? {26a}

Patients diagnosed with one or several diagnoses of chronic psychiatric disorder (schizophrenia, schizoaffective disorder, bipolar disorder, recurrent major depression, borderline personality disorder) or ASD, treated as outpatients or in a day hospital, will be screened for eligibility to participate in this study.

After at least 2 weeks of reflection after receiving study information during a routine visit, patients (or their legal representative) are invited to meet with the research psychiatrist to discuss any remaining questions and sign the informed consent.

### Additional consent provisions for collection and use of participant data and biological specimens {26b}

This trial does not involve collecting biological specimens for storage.

### Interventions

#### Explanation for the choice of comparators {6b}

There are two control groups (active and passive control): The active control group consists of a 12-week treatment of ESS psychoeducation, because recent meta-analysis and systematic review recommended this treatment [30]. The passive control group consists of 12 weeks of treatment as usual (TAU—e.g., visits in the context of a day hospital or as an outpatient). Given the limited number of interventions targeting internalized stigma in France, TAU is the comparator of choice.

### Intervention description {11a}

Participants randomized to the experimental arm and the active control arm will begin the intervention (CFT or ESS) within 1 month, to allow for the formation of a group of 8 individuals for each active arm.

Both therapies (CFT and ESS) will follow the same modality: groups of 8 participants, 12 sessions of therapy facilitated by 2 co-therapists (one senior psychologist and one mental health professional or peer support worker). The duration of each session will be 2 h including a 15-min break. For both therapies, a whiteboard and therapy manuals (booklets for the participants with summaries of key information and worksheets and booklets for the facilitators) will be provided with aims and contents for each session and examples of verbatim for the therapist. Both therapies will include in-between session practices for the participants.

Participants randomized to the TAU arm will pursue their routine care (including visits to their psychiatrist and other treatment modalities delivered in their routine care with the exception of specific psychotherapies, such as CBT).

## Experimental arm (CFT)

CFT is an experiential therapy. As such, in addition to psychoeducation components (e.g., compassion from an evolutionary and neuroscientific perspective, the tricky brain problem, emotion regulation systems) and explicit learning of emotion regulation skills (in particular, shame), experiential exercises are provided in-sessions (e.g., chair work, role plays, guided mental imagery) and in-between session practices will be provided with video guides, made available for the participants online (e.g., soothing rhythm breathing, safe place imagery, compassionate self-imagery). The overall aim of the CFT program is to help participants shift from a hostile and critical self-to-self relationship to a more compassionate relationship to self. To increase compassion for self, others and the ability to receive compassion from others, the CFT therapist guides patients to develop feelings of warmth, safeness, and soothing through compassionate mind training [32]. To do so, an integral part of CFT involves learning about brain functioning, specifically the roles of three emotion regulation systems: (i) the threat detection and protection system, (ii) the drive system, and (iii) the soothing and social safety system [32]. CFT proposes that psychological difficulties arise when the three emotional regulation systems are out of balance. Compassionate mind training strengthens the soothing system which can then regulate the threat and drive systems [52].

## Active control arm (psychoeducation-ESS)

ESS sessions cover topics such as the path from public stigma to self-stigma and modifying self-stigmatizing thoughts through cognitive restructuring techniques. Participants will be encouraged to do home practices (e.g., writing about the pros and cons of self-stigmatizing thoughts) between sessions.

Tables 1 and 2 show the detailed descriptions of CFT and ESS programs.

**Table 1:** Title, content of sessions, and home practices of the CFT program (experimental arm)

**Session number**	**Session title**	**Session content**	**Home practice**
**1**	Welcoming and creating a safe place Definition of compassion and personal goals	• Introducing members of the group and therapists • Collective reflection on a safe place agreement for the group • Exercise: step in/me too • Exploration of what is (and what is not) compassion • Identification of personal objectives for the therapy • Short introducing to soothing rhythm breathing (SRB)	Soothing rhythm breathing (SRB) https://youtu.be/Md2c0h6bogE)
**2**	Compassion wisdom: the tricky brain and the social construction of self	• SRB • Tricky brain problem • How and why we are different to other animals: our unique capacity for self-consciousness and self-judgment (“not our fault”) • We are only one version of the infinite possible versions of self • Understanding the influence of our social environment on our construction (“not our fault”)	Soothing rhythm breathing (SRB) Identifying my own tricky brain loops
**3**	Compassion wisdom: three emotional regulation systems	• SRB • Introducing the three circles model: threat, drive, and soothing • Evolutionary function of emotions	Soothing rhythm breathing (SRB) Drawing my three circles and identifying triggers
**4**	Compassion wisdom: stigma and self-stigma	• SRB • Introduction stigma and self-stigma • Understanding the path from public stigma to self-stigma (“not our fault”) through the social construction of self and the tricky brain • Consequences of self-stigma through the lens of the 3-circle model	Soothing rhythm breathing (SRB) Filling the self-stigma model and tricky brain loops associated
**5**	Compassionate engagement: thinking, imagery, and body postures can influence our physiology	• SRB • Introducing the mindfulness circle • Thoughts and imagination can impact our physiology: experiencing with attention, postures, tones of voice, SRB • Safe place imagery • Ideal compassionate other imagery	Safe place imagery https://youtu.be/Md2c0h6bogE
6	Compassionate engagement: the compassionate self	• Experiencing with the compassionate self (postures, tone of voice, feelings of warmth, actions)	Compassionate self-imagery https://youtu.be/1KELVnBvvho
7	Compassionate courage: multiple selves	• Embodying the compassionate self to respond to the threat system thoughts and emotions	Compassionate self-imagery https://youtu.be/1KELVnBvvho
**8**	Compassionate courage: how to respond to the self-stigmatizing self	• Exploration of self-stigma and self-critic: reasons to be and consequences • Using compassionate self to respond to self-stigma	Compassionate self-imagery https://youtu.be/1KELVnBvvho
**9**	Compassionate courage: dealing with difficult emotions	• Understanding of shame and guilt • Responding to difficult emotions with compassion	Embodying compassionate self in everyday life
**10**	Compassionate courage: compassionate assertiveness	• Understanding the components of compassionate assertiveness compared to submissive and aggressive expression • Practicing compassionate assertiveness through role plays	Compassionately asking something we need
**11**	Compassionate courage: cultivating the compassionate self	• Writing a compassionate letter • Sharing of compassionate letters	Compassionate letter
**12**	Continuing my journey with compassion	• Reflection on the last 12 weeks • Building my personal compassionate tool bag • Plans for continuing practicing compassion • Gratefulness and compassion wish

**Table 2:** Title, content of sessions, and home practices of the pychoeducation-ESS program (active control arm)

**Session number**	**Session title**	**Session content**	**Home practice**
**1**	What is stigma regarding mental illness, where does it come from; what are its impacts?	Self-reflections regarding stigma. Myths and facts about mental illness	Identifying negative ideas and assumptions about mental illness
**2**	What is internalized stigma and what are its impacts?	Self-reflections regarding internalized stigma Common stereotypes and their connections to internalized stigma	Identifying negative ideas and assumptions about myself linked to my mental illness
**3**	“Automatic thoughts” as part of internalized stigma	The 3C’s strategy to ameliorate/challenge them, part 1: catch it	Catching automatic thoughts
**4**	Completing the 3C’s strategy	Part 2: check it and change it. The thoughts behaviors-feelings cycle and internalized stigma	The 3C practice: catch, check, change
**5**	Importance of and strategies for strengthening yourself	“Growing” the positive aspects of yourself that you may have put on the back burner due to mental illness and other challenges	Facets of myself Reclaiming and strengthening parts of my true self
**6**	Increasing belonging	Part 1: the importance of belonging and positive connections with others in staying strong, resisting internalized stigma, enhancing quality of life, and reducing alienation	Adding to belonging: identifying: activity, value, interest
**7**	Increasing belonging	Part 2: practical strategies for increasing belonging	Adding to belonging: next steps
**8**	Increasing belonging	Part 3: the importance of and strategies for optimizing relationships with family and close friends. Increasing the positive, reducing the negative, taking care of oneself to enjoy the former, and tolerate the latter	Increasing belonging with family and friends
**9**	Responding to stigma, disrespect, or discrimination from others	Part 1: in ways that do not lead to internalized stigma, revisiting the thoughts feelings-behaviors cycle	Managing discrimination related to mental illness
**10**	Responding to stigma, disrespect, or discrimination from others	Part 2: cognitive and behavioral strategies	Managing discrimination related to mental illness
**11**	Course recap	Summary of each session and its accompanying strategies	My tools and strategies
**12**	Next steps	Crafting an action plan for after the class. Course evaluation	

## Passive control arm (TAU)

Participants will not participate in any intervention aimed at reducing self-stigma. They will continue their usual treatment which might include weekly visits to their psychiatrist. Participants and therapists in each center will receive, after the study period, access to the ESS notebook.

### Criteria for discontinuing or modifying allocated interventions {11b}

Participants may withdraw their consent and request to leave the study at any time for whatever reason. The investigator must document the reasons for early discontinuation as fully as possible. The investigator has the right to discontinue a subject’s participation in the study, temporarily or permanently, for any reason that is in the best interest of the subject.

Participants who prematurely discontinue the study procedure will continue follow-up in the study until the end-of-study visit scheduled in the protocol. The criteria for premature termination of participation by the investigator are long-term hospitalization (more than 1 month).

For patients in the three arms, in case of premature termination of participation in the study by decision of the investigator or the patient/guardian, the self-report questionnaires including the ISMI will be administered one last time to the exiting participant, if the patient/guardian agrees. A semi-structured evaluation interview will also be conducted.

### Strategies to improve adherence to interventions {11c}

Supervision sessions will be held monthly with the Strasbourg team to increase adherence to the psychotherapy models. Moreover, ESS clinicians will not deliver CFT (and vice versa) in order to increase adherence.

Members of the Strasbourg team will participate in monthly supervision sessions with the developers of ESS and group CFT expert clinicians to increase adherence.

Treatment fidelity checks (via fidelity tracking sheets) will be conducted by observers (e.g., graduate-level psychology students) to ensure reliable delivery of treatment and make further replication possible.

### Relevant concomitant care permitted or prohibited during the trial {11d}

During the trial, all concomitant treatments are accepted except CFT and CBT (including psychoeducation) for self-stigma. A participant starting psychotherapy in parallel to their participation in the protocol will be kept in the group for ethical and clinical reasons. Their data will be excluded from the study.

### Provisions for post-trial care {30}

Strasbourg University Hospital, the sponsor of the study, has taken out an insurance policy for the entire duration of the study that covers its own public liability as well as that of any person involved in the conduct of the trial regardless of the nature of the relationship that exists between these persons and the sponsor.

### Outcomes {12}

#### Primary outcomes

The decrease in scores on the Internalized Stigma of Mental Illness Scale (ISMI) [31] will be our primary outcome. The ISMI scale has good psychometric qualities in its various language versions [20]. This measure is a 29-item self-report questionnaire with five subscales: (1) alienation, (2) endorsement of perceived stereotypes, (3) discrimination, (4) social withdrawal, and (5) resistance to stigma. The participant must select their response on a 4-point Likert scale ranging from strongly disagree (1) to strongly agree (4).

The ISMI scale will be administered before therapy (V0), at the end of therapy (V1), and 6 months after the end of therapy (V2). If CFT is efficacious in reducing self-stigma, ISMI scores will decrease significantly between V0 and V1, and this decrease will be greater for CFT than for psychoeducation (ESS) and TAU.

### Secondary outcomes

Assessment of the maintenance of self-stigma scores:

• To evaluate the maintenance of effects on *self-stigma* in the three groups of treatment (CFT, psychoeducation, and TAU) at 6-month follow-up (V2) the Internalized Stigma of Mental Illness Scale (ISMI) [31] will be applied.

Assessment of the effects of treatment (CFT, psychoeducation, and TAU) at baseline (V0), post-treatment (V1), and at 6 months follow-up (V2) on target psychological dimensions and assessment of the psychological factors mediating the efficacy of CFT on the self-stigma measure:

- The Internalized and Externalized Shame Scale (EIS) [53] will be used to measure shame. It consists of 9 items ranging from 1 (not at all) to 4 (a lot).
- The Phenomenological Body Shame Scale – Revised (PBSS-R) [54] will also be used to measure body-related shame. Each of the 8 items is scored on a scale ranging from 1 (not at all) to 5 (extremely).
- The Self-Compassion Scale (SCS) [55] will be applied to measure self-compassion. The 26 items are scored on a scale ranging from 1 (almost never) to 5 (almost always).
- The Difficulties in Emotional Regulation Scale – 16 items version (DERS-16) [56] will be used to measure emotional regulation competencies. Each item is scored on a scale ranging from 1 (almost never) to 5 (almost always).
- The automatic avoidance tendencies toward mental disorders (VAAST) [57, 58] assesses the participants’ automatic avoidance tendencies toward mental disorders. The VAAST simulates the approach and avoidance movements of the whole self by manipulating the visual information provided to the participants. A stimulus first appears in the center of the screen in a simulated street background. Participants have to press the “move toward” or the “move away” key as a function of the stimulus category and the instructions. This complementary measure allows to circumvent some biases (such as social pressures) in the responses to the self-report scales.

Assessment of the effects of treatment (CFT, psychoeducation, and TAU) at baseline (V0), post-treatment (V1), and at 6 months follow-up (V2) on social functioning and psychiatric symptoms:

• The Brief Psychiatric Rating Scale (BPRS) [59, 60] will be used to assess psychiatric symptoms. The BPRS is a rating scale which a clinician or researcher may use to measure psychiatric symptoms such as depression, anxiety, hallucinations, psychosis, and unusual behavior. The rater enters a number for each of the 24 symptom constructs that ranges from 1 (not present) to 7 (extremely severe).
• The Depression, Anxiety, and Stress Short Scale – 21 items version (EDAS 21) [61, 62] will be applied to measure depression, anxiety, and stress. Each item is scored on a scale ranging from 0 (did not apply to me) to 3 (applies to me very much, or most of the time).
• The Social Functioning Questionnaire (SFQ) [63] will be used to measure social functioning. It consists of 16 items divided into two subscales: (1) the frequency of occurrence for different activities/events and (2) the participants’ satisfaction regarding said activities/events. Items of subscale 1 are rated from 0 (never) to 1 (everyday), and items of subscale 2 are rated from 0 (very unsatisfied) to 4 (very satisfied).
• The Barriers to Accessing Care (BACES-EN-VI) [64] will be applied to assess participants’ access to care. Only the subscale concerning barriers to accessing care related to the stigma of mental illness will be used. The 12 items of the subscale are scored using a scale ranging from 0 (not at all) to 3 (a lot).
• The Recovery Assessment Scale – short version (RAS) [65, 66] will assess participants’ recovery from the disorder. It consists of 20 items ranging from 1 (strongly disagree) to 5 (strongly agree).
• The Functional Remission of General Schizophrenia Scale (the mini-FROGS) [67] will assess functional remission. The clinician-researcher will rate the 4 items, using a scale ranging from 1 (does not do it) to 5 (does it perfectly).

To evaluate the *acceptability* of the active interventions (CFT and ESS):

• The Credibility and Expectations Questionnaire [68, 69] will be applied to assess self-reported treatment credibility at V0. This questionnaire contains 6 items rated either from 1 to 9 or from 0 to 100% and evaluates whether the participants “think” or “feel” that their treatment will be efficient.
• The Group Questionnaire [70] will be used to measure group cohesion 3 times during the intervention (sessions 1, 6, and 12). It consists of 30 items addressing the group leaders, the other group members, and the group in general. Each item is scored using a scale ranging from 1 (not true at all) to 7 (very true).
• The Consumer Satisfaction Questionnaire for Psychotherapeutic Services (CSQ-8) [71] will be applied as a self-report measure assessing participants’ satisfaction at the end of the 12-week program. The 8 items are scored on a scale ranging from 1 to 4. Higher scores are indicative of elevated satisfaction.
• The attendance rate will be determined by the number of group sessions attended by the patient, and the drop-out rate will be determined by the number of patients lost to follow-up in each arm. In addition to the Consumer Satisfaction Questionnaire for Psychotherapeutic Services questionnaire (CSQ 8) [71], the avoidance of participants (due to self-stigma) in several social situations during the last 6 months, and the satisfaction with the interventions will be assessed via semi-structured interviews conducted at V2 by clinician-researchers blind to the status of patients (in the Strasbourg center only, for the 3 groups—CFT, ESS, and TAU).

### Participant timeline {13}

#### Information briefing (> D7 before V0)

The investigator (psychiatrist) will offer patients the opportunity to participate in the study during a routine consultation, providing full details about the study procedure: the nature of the study, the objectives of the therapy, and the reasons for the indication. They will give the information leaflet entitled “Information leaflet for adult patients” to the patient which consists of information on the trial, with a consent form. For patients under guardianship, information adapted to their capacity to understand will be given. If the guardian is absent during this visit, the study information document can be sent to them by e-mail or by post by the physician. The guardian will be invited to accompany the patient to the inclusion visit so that the investigator can give them the information in person and answer any questions they may have. Consent may be signed by the guardian prior to the inclusion visit (V0), if the guardian agrees to the person’s participation. The patient/tutor/guardian will be informed that they will not be allowed to participate in an interventional study involving psychotherapy or an experimental drug for the duration of their participation in the protocol. The patient/tutor/guardian will be informed about the importance not to disclose their assigned treatment group to the psychiatrist involved in the recruitment process during their participation in the study, in order to keep the psychiatrist blinded for post-treatment evaluations using the BPRS and the Mini-FROGS. The patient/tutor/guardian is free to accept or reject the offer to participate. They will be given sufficient time (7 days minimum) to think over their decision.

### Inclusion visit (V0)

The first appointment at the hospital center is organized with the person (and the tutor/guardian, if applicable) to provide all necessary information about the intervention. The investigator answers all the questions the patient might have. The eligibility of the patients (inclusion and non-inclusion criteria) will be carried out by the psychiatrists in each center. The following routine evaluations will be conducted: intellectual disability estimated via the fNART, severity of self-stigma estimated by the ISMI, and severity of mental illness via CGI score.

Individuals with scores as indicated below will be included in the study; an inclusion number will be allocated to the patient:

• ISMI self-questionnaire score ≥ 2
• IQ > 70
• CGI-Severity score < 6

Following this and if they meet the eligibility criteria and they agree to participate in the study, the patient may sign the consent form. The consent of the subject under guardianship will also be sought to the extent that their condition permits, and their refusal or revocation of acceptance will not be overridden. The patient/tutor/guardian will be given a copy of the informed consent.

The date on which the person agrees to participate in the research is noted in their medical record, as well as the date on which they object to participation, if applicable.

### Randomization

The investigator (psychiatrist) will then confirm the eligibility of the patient to another investigator (psychologist or nurse) of their team, who will be in charge of the randomization of the patients on the CleanWEB platform. This will ensure a blind assessment of the post-treatment evaluation by the psychiatrist at V1 and V2 (i.e., the BPRS and Mini-FROGS scales).

Patients will be randomized by the psychologist or the nurse into one of the following arms:

- Experimental arm: 12 week-treatment of compassion-focused therapy (CFT) (i.e., psychoeducation on the model, experiential in-session exercises, and in-between session compassion practices, video-guided practices). Sessions cover several topics: psychoeducation on compassion from an evolutionary and neuroscientific perspective, emotion regulation skills (especially shame), and compassion practices.
- Active control arm: 12 week-treatment of the psychoeducation program *Ending Self-Stigma* (ESS) [29]. Sessions cover topics such as modifying self-stigmatizing thoughts and homework.
- Passive control arm: patients follow their usual care, i.e., TAU (e.g., psychiatric, psychological, day hospital), which will be unchanged during the 12-week period. Following their 10-month participation in the study, they will be given access to the written material used for the ESS.

Participants will then be asked to complete self-report questionnaires assessing self-compassion, shame, depression, anxiety, barriers to care, social functioning, recovery, and functional remission. During this visit, the cognitive task assessing avoidance/approach attitudes toward mental illness (VAAST) will also be carried out by the psychologist or the nurse.

Two individual sessions with a clinician (engagement phase) will be conducted between V0 and the beginning of CFT and ESS (i.e., within 1 month). These sessions will include motivational interviewing techniques and case conceptualization aimed at increasing the participant’s level of engagement in therapy.

### Experimental or control therapies (12 weeks)

Participants randomized to the experimental arm and the active control arm will begin the intervention (CFT or ESS) within 1 month, to allow for the formation of a group of 8 individuals for each active arm. Participants randomized to the TAU arm will pursue their routine care.

Both therapies (CFT and ESS) will follow the same modalities: groups of 8 patients, facilitated by 2 co-therapists (one senior psychologist and one mental health professional or peer support worker). Sessions will have a duration of 2 h including a 15-min break, therapy manuals and a whiteboard will be used and both therapies will include homework practices.

### Follow-up visit V1 (end of treatment + 1 week)

This visit corresponds to the end-of-treatment assessment (T1) for patients in each arm, starting at week 18 after inclusion (following the last group session for the active arm and the active control arm). This evaluation includes the administration of the self-report questionnaires, including the ISMI, and the administration of the cognitive task (VAAST). A psychiatrist blinded to the experimental arm of the patient will conduct an evaluation of psychiatric symptoms and functional remission via the BPRS and the Mini-FROGS.

### Follow-up visit V2 (end of treatment + 6 months)

This visit corresponds to the follow-up assessment (T2) for patients of each arm, starting 6 months after the last group treatment session (and 9 months after the inclusion of the TAU group). This evaluation includes the administration of the self-report questionnaires, including the ISMI, the administration of the cognitive task (VAAST). A psychiatrist blinded to the experimental arm of the patient will conduct an evaluation of psychiatric symptoms and functional remission via the BPRS and the Mini-FROGS**.**

Moreover, in the Strasbourg center only, a semi-structured interview will be led by a clinical psychologist blinded to the experimental arm of the patient (i.e., a clinical psychologist not involved in the trial to make sure that they had no previous contact with the participant). The interview will assess the avoidance of participants (due to self-stigma) in several social situations as well as their satisfaction with the intervention (CFT and ESS).

**Fig. 1:**
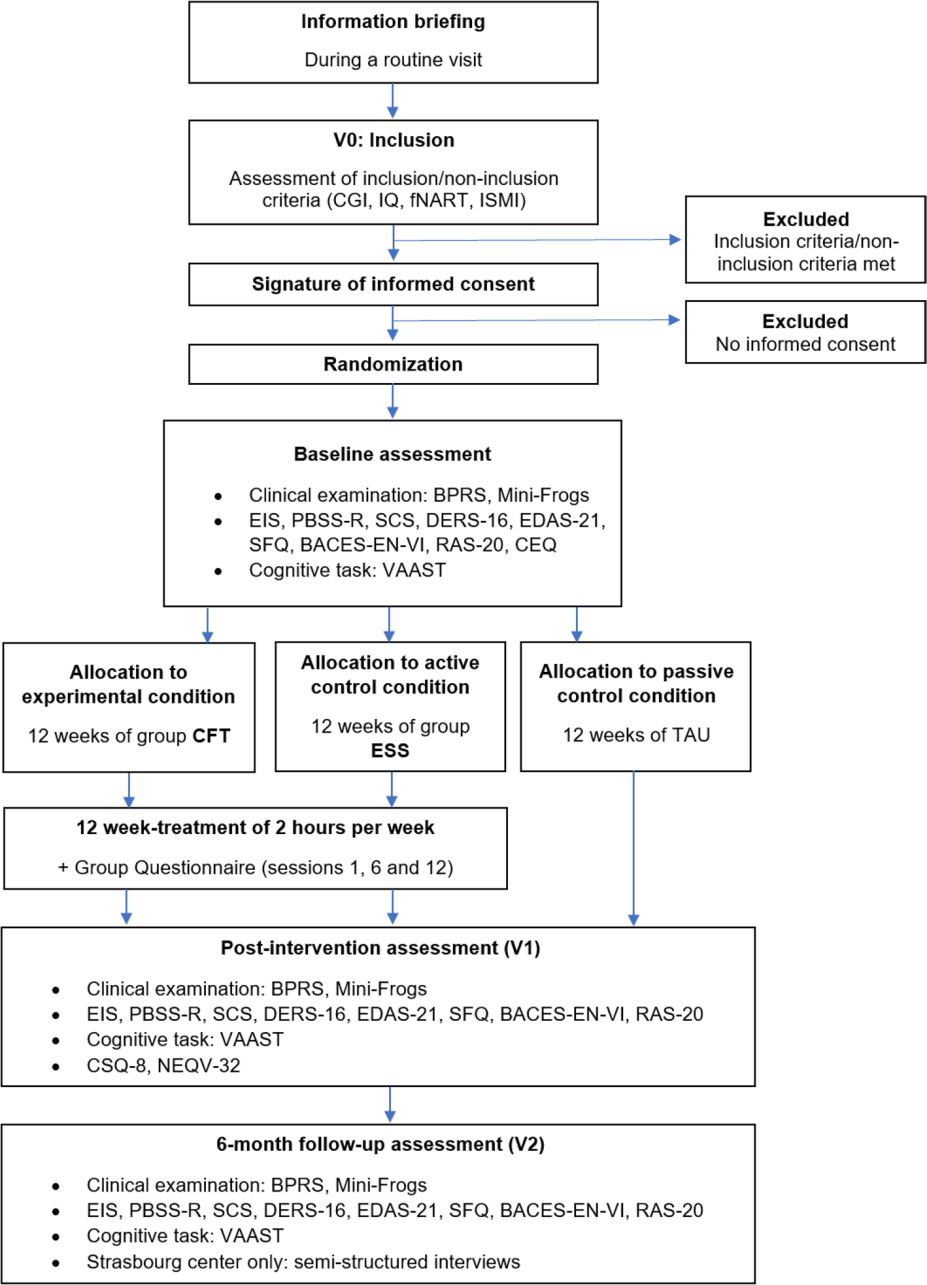
Flow chart of the COMPASS study

An additional 1-month window will be allowed to conduct this visit. The participation of patients ends at the end of this visit (Fig. 1). Table 3 shows the schedule for the COMPASS study.

**Table 3:**
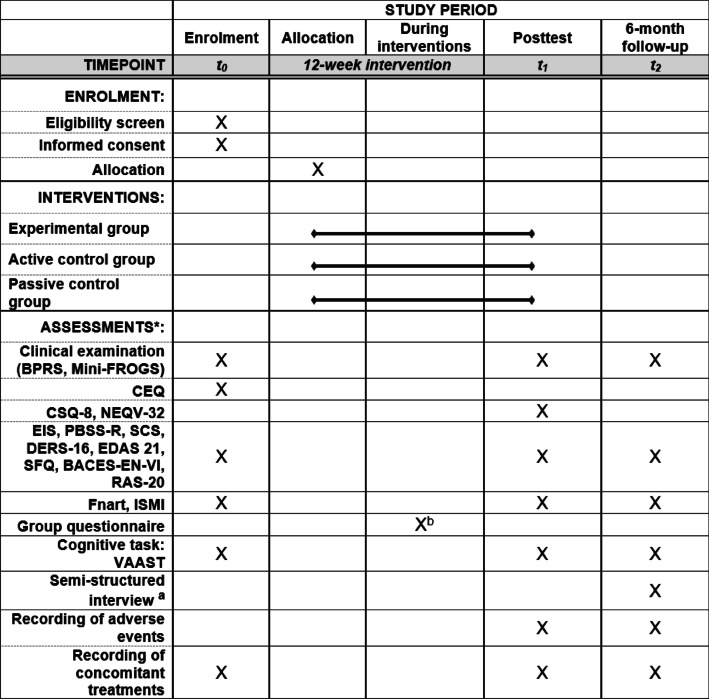
Schedule for the COMPASS study

### Sample size {14}

Taking the effects obtained in the article *Effects of a group psychoeducation program on self-stigma, empowerment and perceived discrimination of persons with schizophrenia* [72], for the TAU and ESS groups and adding a CFT group with a stronger and lasting effect, the means and standard deviations of the ISMI before the therapy, after the therapy, and after 6 months follow-up used to estimate the sample size are respectively 2.46 ± 0.39, 2.43 ± 0.29, and 2.46 ± 0.39 for the TAU group; 2.45 ± 0.48, 2.20 ± 0.41, and 2.40 ± 0.48 for the psychoeducation group; and 2.45 ± 0.48, 2.10 ± 0.41, and 2.20 ± 0.48 for the CFT group. With 84 patients in each group, the power is more than 85% between the TAU and psychoeducation groups after the therapy and more than 99% between CFT and the other groups after 6 months. In order to consider the possible loss to follow-up, the sample size is increased by 33% to obtain 112 patients in each group. The total sample size is therefore 336 participants.

### Recruitment {15}

A large network of professionals in institutions or in private practice (e.g., psychiatrists, psychologists) of the geographical area of all the participating centers of the study will be deployed to refer patients to the investigating teams. All the hospital centers are central coordinators of the care of patients with SMI and ASD in their respective areas; moreover, all of them propose care within a recovery model of psychiatric disorders. These factors guarantee that many patients with SMI and ASD can be recruited in these centers and that all centers will show high levels of implication in the recruitment and adherence to the psychotherapeutic models proposed.

Patients will be recruited into the psychiatric and mental health department of each center during a routine clinical consultation. The inclusion period is 48 months. This is a realistic duration given the significant number of patients to be enrolled. The study is scheduled to involve 7 French national centers, with 24 patients to be enrolled per center (one randomization blocks of 24 patients—3 groups of 8 patients in CFT, ESS, and TAU—in each center; the Strasbourg center will recruit 192 patients, i.e., 8 randomization blocks).

### Assignment of interventions: allocation

#### Sequence generation {16a}

Randomization will be done through the CleanWEB® platform, which will be accessed by the investigator or a designated person, using their personal access codes. The investigating team and the sponsor will receive an email confirming the randomization. Patients will be randomized over a period of 1 month before the start of the interventions, to allow the 3 groups of treatment to be formed (CFT, ESS, TAU), so that each group starts the allocated therapy at the same time.

The randomization will be stratified by center, with a 1:1 ratio for allocation to the 3 groups. Patients will be recruited until they form a group of 24 people allowing to randomize them in one of the 3 conditions, in groups of 8 people matched on gender and diagnostic group (4 possibilities: (1) psychotic disorder, (2) mood disorder, (3) personality disorder, and (4) autism spectrum disorder, referring to (1) schizophrenia-spectrum disorder, (2) bipolar disorder and depression, (3) borderline personality disorder, and (4) autism spectrum disorder). This process will be reiterated at least once in each center:

### Concealment mechanism {16b}

Concealment will be ensured as the CleanWEB® platform will not release the randomization code until the patient has been recruited into the trial.

### Implementation {16c}

The allocation sequence is configured by the data manager who is blinded to the allocation group. Participants will be enrolled and assigned to interventions by investigators of the center team (different from the psychiatrist performing the clinical examination).

### Assignment of interventions: blinding

#### Who will be blinded {17a}

Participants will be informed of the group which they are allocated to. Care providers are informed of which treatment group they are facilitating. The psychiatrist performing the clinical examination and assessing the outcomes (at V0, V1, V2) is not involved in the randomization process and remains blind to the groups to which patients are attributed throughout the trial. The allocation sequence is configured by our data manager who is blinded to the allocation group.

In the Strasbourg center only, the clinical psychologist administrating the semi-structured interview will be blinded to the experimental arm of the patient (i.e., a clinical psychologist not involved in the trial to make sure that they had no previous contact with the participant).

### Procedure for unblinding if needed {17b}

The design is open-label with only outcome assessors and the clinical psychologist performing the semi-structured interviews being blinded so unblinding will not occur.

### Data collection and management

#### Plans for assessment and collection of outcomes {18a}

Self-report questionnaires will be completed on a survey software (Limesurvey) using a tablet computer at V0, V1, and V2, in order to increase the ease of use for participants.

Clinician-reported data will be collected on the online survey software (CleanWeb) by the investigators and will be stored on a secure hospital server.

The assessors will be trained to use the study instruments prior to the inclusion phase. The respective measurement tools that will be applied are described in the outcomes section {12} including information on reliability and validity. In order to promote data quality, we only included validated scales.

### Plans to promote participant retention and complete follow-up {18b}

Patients enrolled in the study will receive a compensation (i.e., 100 euros) at the end of the last visit with the psychiatrist. To receive the compensation, participants must have completed the visits V1, V2, and V3.

For patients in the three arms, in case of premature termination of participation in the study by decision of the investigator or the patient/guardian, the self-report questionnaires including the ISMI will be administered one last time to the exiting participant, if the patient/guardian agrees. A semi-structured evaluation interview will also be conducted. For patients who quit the study prematurely, all data collected before the decision to interrupt the study will be analyzed.

A participant starting psychotherapy in parallel to their participation in the protocol will be kept in the group for ethical and clinical reasons. Their data will be excluded from the study.

### Data management {19}

All study data will be anonymized and then entered into an electronic case report form by the investigator or a person appointed by the investigator. A record of all changes made to the case report forms will be retained. This record must make it possible to ascertain for any given change the previous value, the date of the change, and the person who made the change. In accordance with ICH-GCP part 11, the investigator will append his or her signature to validate the authenticity and accuracy of the data in the case report form and the signature being electronic in electronic case report forms.

The database connected to the CleanWEB® electronic case report forms is hosted by TELEMEDICINE Technologies S.A.S., which possesses a number of dedicated virtual data centers. This company ensures data confidentiality, security, and integrity in accordance with a safety plan predefined by the sponsor and compliant with international recommendations (ICH-GCP part 11).

Trial data will be managed using the CleanWEB® software solution marketed by TELEMEDICINE Technologies S.A.S.

The list of people authorized to access the data is established by the project lead (coordinating/principal investigator) and the sponsor. Each person will be given strictly private, confidential login details to access the CleanWEB® input masks.

The coherence of the data collected will be verified by computer according to rules predefined between the sponsor and investigator and described in the monitoring plan. Queries will be sent to the investigator to clarify or correct certain data items. Every change will be traced via an audit trail, which may be consulted using CleanWEB®.

### Confidentiality {27}

The principal investigator will ensure that patient anonymity is observed and retains a confidential list identifying the patients included. In accordance with Article R.5121-13 of the French Public Health Code, the investigators and all those who are to work on the trial are bound by professional secrecy with regard in particular to participants and the results obtained. Without the sponsor’s consent, they may give information relating to the trials to the health minister, public health physicians, public health pharmacists, or the director and inspectors of the French National Agency for Medicines and Health Products Safety only.

The processing of personal data used within the framework of the study will be carried out according to the conditions laid down by Law 78-17 of January 6, 1978 (as amended) relating to information technologies, data files and civil liberties, and the regulations established to implement it, and by the European Directive UE 2016/679 dated 27 April 2016 related to personal data protection.

The data processing performed within the framework of this study will be carried out in accordance with all the provisions of reference methodology MR-001 on data processing in clinical trials as updated on 3r May 2018, by decision 2018-153 of the CNIL. Strasbourg University Hospital signed an MR-001 compliance statement on January 8, 2009.

### Plans for collection, laboratory evaluation, and storage of biological specimens for genetic or molecular analysis in this trial/future use {33}

N/A: no collection, laboratory evaluation, and storage of any biological specimens for genetic or molecular analysis in this trial/future use.

## Statistical methods

### Statistical methods for primary and secondary outcomes {20a}

The statistical analysis will have descriptive and inferential. The analyses will be carried out according to Bayesian methods.

The descriptive statistical analysis of the categorical variables will be performed by giving the frequencies, the cumulative frequencies, the proportions, and the cumulative proportions of each value. Whenever useful, cross-tabulations will be given with frequencies, proportions by row, proportions by column, and proportions of the total for each box in the table. For each continuous variable, the location parameters (mean, median, minimum, maximum, first and third quartiles) and the dispersion parameters (variance, standard deviation, range, interquartile range) will be given. The continuous variables will be described with histograms (with different bin width and with kernel density estimation). The normality of the distributions will be tested using a normality test, such as the Shapiro-Wilk test, and will be assessed graphically using a normal quantile plot.

To fulfill the primary objective, a generalized Bayesian linear regression model will be performed in order to estimate the effect of the treatment considering the center and the subject as a random effect. According to the distribution of the primary endpoint, i.e., the ISMI, the linear regression will be Gaussian, beta, or gamma.

To fulfill the secondary objectives, a generalized Bayesian linear regressions model will be performed: binary variables will be analyzed with a Bayesian logistic regression, score variables will be analyzed with a Bayesian beta regression, count variables will be analyzed with a Bayesian Poisson regression, and continuous variables will be analyzed with a Bayesian linear regression.

Priors will be firstly weakly informative and secondly more informative in a sensitivity analysis.

For each analysis, the posterior distribution of the parameter of interest (rate, proportion, mean, odds-ratio, regression coefficient, etc.) will be estimated using Markov chain Monte Carlo (MCMC) draws. The default number of iterations is 100,000 with a burn-in of 10,000 and a thinning of 2 on each of 3 chains. Algorithm convergence will be assessed graphically and with the Gelman-Brooks-Rubin (GBR) test. Autocorrelation will be assessed graphically, and if required, the number of iterations and thinning will be increased to reduce as much as possible the autocorrelation.

The mechanisms underlying the relationship between the intervention and the primary endpoint will be identified by carrying out an estimation of the causal mediation effect using the Baron and Kenny procedure though the quasi-Bayesian approximation. This allows for obtaining total, direct, indirect effects and the percentage of mediation. The potential mediators that will be tested include self-compassion, shame, and emotional dysregulation.

The statistical analyses will be run with the following software: R (with all the relevant packages), OpenBUGS, and JAGS, in their most up-to-date version at the time of analysis.

There is no significance level in Bayesian statistics. An effect will then be considered significant if its probability to exceed a prespecified threshold value, given the data, is strictly larger than 0.975 or strictly lower than 0.025. Credibility intervals will be provided at 95%.

### Lexical/semantical analyses

The data obtained via 48 semi-directed interviews (the first two samples recruited in Strasbourg) will be analyzed using the software Iramuteq 0.7 (R interface for multidimensional analysis), which is designed to perform lexical/semantical analysis. First, we will extract the most listed characteristics, independently of grammatical form. Second, we will analyze specificities for each sub-group of patients. Third, to check if these specificities have a statistical meaning, we will extract relative occurrences (occurrences depending on the total number of words produced) for these characteristics and compare the number of relative occurrences for each type of subgroup, using the chi^2^ test.

### Interim analyses {21b}

Stopping of the trial could also be based on interim data analysis if clearly one treatment is better than the other, but no interim analysis is planned.

### Methods for additional analyses (e.g., subgroup analyses) {20b}

No additional analyses are planned.

### Methods in analysis to handle protocol non-adherence and any statistical methods to handle missing data {20c}

Subjects with at least one non-missing data, for a given analysis, will be included in the analysis.

In order to minimize attrition bias, an intention-to-treat analysis and specific techniques for handling missing data will be performed.

Missing data frequency will be listed for every variable. A univariate and bivariate (i.e., for each relevant group) description will be provided. Monotone patterns will be looked for and a probable missing data mechanism (completely at random, at random, or not at random) will be identified.

Missing data will be treated according to their frequency in a given analysis. Simple deletion will be considered if the proportion of missing data is smaller than 3%. For a missing data proportion between 3 and 20%, multiple imputations will be used. For larger proportions of missing data, the relevance of each analysis will be discussed and, if required, specific models will be used.

Multiple imputations will be implemented and run directly within the Bayesian model scripts.

### Plans to give access to the full protocol, participant-level data, and statistical code {31c}

The consent form, information material, and database are available from the corresponding author on request.

### Oversight and monitoring

#### Composition of the coordinating center and trial steering committee {5d}

The coordinating center in Strasbourg will be led by MR, SW, and LW and include 2 additional psychologists. Their role is to supervise the inclusion process (communication, information of potential recruits, consent) and to explain and guide the other centers in their own inclusion and assessment process, providing standardized documents for the inclusion call, communication means, and data storage. The psychotherapy coordinating team (LW, MR) will also hold monthly supervision sessions to increase adherence to the psychotherapy models. This coordinating group will meet weekly. A scientific committee, composed of the main investigator (LW), associated scientific member and investigator (MR), the methodologist (LF), and a promoter representative, wrote the protocol, selected the investigators, and will decide whether to modify, continue, or stop the project.

### Composition of the data monitoring committee, its role, and reporting structure {21a}

A data manager configured the online survey software (CleanWeb) for hetero-reported data and the investigator (MR) configured the online survey for self-reported questionnaires (Limesurvey). They made sure data storage was secure. Any modification will be referred to the ethics committee (Comité de Protection des Personnes - Nord Ouest II).

### Adverse event reporting and harms {22}

According to article L1123-10 of the French Code of Public Health (*Code de la Santé Publique*), any adverse reaction/incident will be reported through the Ministry of Health’s Adverse Health Event Reporting Portal. These events will be reported in the case report form, from the time that written informed consent is given, up until the end of the subject’s participation in the study.

### Frequency and plans for auditing trial conduct {23}

All data, documents, and reports may be audited or inspected. The investigators undertake to comply with the requirements of the sponsor and of the competent authority with regard to any audit or inspection of the study. The audit may pertain to any stage of the study, from the development of the protocol to the publication of the results, as well as to the binning of data used or produced within the framework of the study.

A clinical research associate delegated by the sponsor may visit each study center while the trial is being launched, once or several times during the trial depending on the inclusion rate, and at the end of the trial. Depending on the level of monitoring set out in the monitoring plan, these visits are carried out to check adherence to the protocol, verify informed consent, check the reporting of serious adverse events and recording of adverse events, and ensure quality control by comparing data in the case report form against the subject’s source documents.

### Plans for communicating important protocol amendments to relevant parties (e.g., trial participants, ethical committees) {25}

In accordance with Article L1123-9 of the French Public Health Code, all substantial modifications will give rise to an application for a binding opinion from the IRB and an application for authorization from the competent authority. Any modification of the protocol, of the instructions, or of the consent form will be referred to the ethics committee (*Comité de Protection des Personnes - Nord Ouest II*). Modified and approved versions of these documents will be transmitted by the promoter to the study coordinating team and to the French agency for health products and medications safety (*Agence Nationale de Sécurité du Médicament et des produits de santé (ANSM)*). The promoter will update the information about the trial on ClinicalTrials.gov.

### Dissemination plans {31a}

Trial results will be communicated at international meetings and via publication of the findings. Trials results will also be communicated to healthcare professionals during hospital meetings and national psychiatry conferences. At the end of the trial, we will also organize an online communication of the results for the participants.

## Discussion

To the best of our knowledge, ours will be the first multi-centered randomized controlled trial evaluating the efficacy and the acceptability of a group-based CFT program on decreasing the self-stigma associated with severe mental illness and ASD. To do so, we will compare a 12-week group CFT program to a psychoeducation program for self-stigma (Ending Self-Stigma) and to treatment as usual (TAU).

Several programs targeting self-stigma have been studied, but given their methodological pitfalls, e.g., lack of randomized controlled studies, lack of sustainability of effects, and low attendance rates, recent studies have emphasized the need to devise innovative approaches [27]. Our trial aims at responding to these gaps in the literature by conducting a large-scale multi-centered randomized trial comparing an innovative intervention for self-stigma to ESS and TAU. ESS was chosen as our active control group because it has been evaluated via two randomized trials and is widely used as routine practice across the world [12, 29]. The results of the first RCT comparing ESS to TAU indicate no improvement in the total self-stigma score after the intervention nor at the 6-month follow-up [29]. There was however an immediate improvement in the alienation and stigma resistance subscales of the Internalized Stigma of Mental Illness Scale (ISMI) [31], but the effects were modest and were no longer significant at 6 months of post-treatment follow-up. In addition, the authors reported a very high drop-out rate and low attendance [29]. The second RCT, comparing ESS to an active control group (non-specific health and well-being program), found no superiority of ESS [12]. Because ESS only targets self-stigmatizing thoughts and behaviors but does not target shame, we hypothesize that the latter might explain the high rates of drop-out and lack of immediate and long-term efficacy of the treatment on reducing self-stigma. This is why CFT, which has been shown to reduce shame across a wide range of clinical populations, should lead to better self-stigma outcomes than the ESS and TAU [73]. In particular, we hypothesize that changes in shame will mediate the relationship between the intervention and self-stigma outcomes.

Given the numerous negative outcomes associated with the self-stigma of mental illness, our findings could significantly improve the well-being, the physical health, and the social inclusion of people with SMI and/or ASD.

Indeed, if our 12-session group program produces a significant decrease in self-stigma and shame scores and improves participants’ social inclusion in a long-term perspective, it could become an empirically assessed viable option specifically suited to the needs of people presenting with SMI or ASD, whose quality of life is diminished due to high self-stigma. Moreover, in terms of format and content, this 12-session-manualized-group CFT has many advantages. Indeed, by targeting key transdiagnostic psychological processes—i.e., self-stigma and shame—it can increase the feasibility and dissemination of the intervention to a high number of clinical contexts (e.g., community health centers, different clinical populations).

Our study protocol presents some limitations. First, the distribution of diagnostic categories might present some inevitable variability across different hospital centers involved. However, our purpose is to propose a transdiagnostic intervention. Relatedly, facilitators’ backgrounds will vary depending on the centers, with possibly different training backgrounds and clinical experiences. To account for this limit, all group facilitators will be trained by the coordinating center clinical team during a 3-day-training specific to each intervention. Second, though concomitant psychotropic medications as well as the use of adjunctive therapeutic means (e.g., self-help or counseling) will be controlled in our study, participants will not be instructed to avoid making use of them. While this might be a potential confound in the current study design, the restriction of therapeutic options would also pose an ethical concern, reduce referrals to the study and also be a threat to external validity. Therefore, by monitoring therapeutic (i.e., medication or counseling) use throughout the trial, the current study seeks to balance the internal and external validity and improve the feasibility of the study while efficiently addressing its hypotheses.

If our innovative group CFT program can effectively reduce self-stigma and shame, it may facilitate the social inclusion of a large number of people with severe mental illness and/or ASD and significantly improve their quality of life and their physical and mental health by reducing barriers to access care.

## Trial status

This is the first version of the protocol (March 1, 2022). Study enrollment has not started yet and will begin in April 2023. The active treatment phase is expected to be finished in December 2024, and completion of follow-up assessments is expected in August 2025. The total sample of 336 participants will be completed by August 2025.

## Data Availability

The study protocol has been reported in accordance with the Standard Protocol Items: Recommendations for Clinical Interventional Trials (SPIRIT) guidelines. The consent form and information material are available from the corresponding author on request.

## Abbreviations

SMI: Severe mental illness
ASD: Autism spectrum disorder
CBT: Cognitive-behavioral therapy
ESS: Ending Self-Stigma
CFT: Compassion-focused therapy
TAU: Treatment as usual
ISMI: Self-Stigma Scale
EIS: Internalized and Externalized Shame Scale
PBSS-R: Phenomenological Body Shame Scale - Revised
SCS: Self-Compassion Scale
DERS-16: Difficulties in Emotional Regulation Scale – 16 items version
VAAST: Automatic avoidance tendencies towards mental disorders
BPRS: Abbreviated Psychiatric Rating Scale
EDAS 21: Depression, Anxiety and Short Stress Scale – 21 items version
SFQ: Social Functioning Questionnaire
BACES-EN-VI: Barriers to accessing care
RAS-20: Recovery Assessment Scale – 20 items version
Mini-FROGS: Functional Remission of General Schizophrenia Scale
CEQ: Credibility and Expectations Questionnaire
GQ: Group Questionnaire
CSQ-8: Consumer Satisfaction Questionnaire for Psychotherapeutic Services – 8 items version
NEQV-32: Negative Effects Questionnaire – 32 items version
CGI: Clinical Global Impressions Scale
fNART: French national adult reading test
